# Trends in Survival and Short-Term Outcomes Among Preterm Infants Less Than 32 Weeks From 2016 to 2022: A Retrospective Cohort Study at a Tertiary Medical Center in the UAE

**DOI:** 10.7759/cureus.103736

**Published:** 2026-02-16

**Authors:** Fahad Butt, Nisha Viji Varghese, Javeria Bhatti, Zohra Siwji, Nusrat Khan, Mustafa Alabdullatif

**Affiliations:** 1 Neonatology, Tawam Hospital, Al Ain, ARE

**Keywords:** bronchopulmonary dysplasia (bpd), chronic lung disease of prematurity, intra-ventricular hemorrhage (ivh), necrotizing enterocolitis (nec), neonatal intensive care unit (nicu), neonatal prematurity, neonatal sepsis, prematurity complications, pvl, survival outcomes

## Abstract

Introduction

Contemporary data on preterm infant outcomes are vital for improving healthcare resources and results, as well as guiding professionals and families in counselling and decision-making.

Objective

To examine the survival rates, in-hospital complications, and antenatal care practices for infants born before 32 weeks of gestational age.

Methods

Demographics and outcome data were retrospectively gathered, adhering to standard definitions, for a cohort of infants born before 32 weeks of gestational age admitted between January 2016 and December 2022 at Tawam Hospital, UAE.

Results

Of the 700 infants with gestational ages 22+0 to 31+6, over the seven years, 553 (79%) survived to discharge. Overall, the median (IQR) length of stay in survivors was 67 (42, 95) days. Bronchopulmonary dysplasia (BPD) (any grade) was 228 (40.5%), and 89 (12.9%) preterm infants had necrotizing enterocolitis (NEC) stage 2 and above. Late-onset sepsis was seen in 195 (30.1%) cases. Regarding neurological injury, intraventricular hemorrhage (IVH) grade 3 and above was recorded in 57 (9.2%) cases, and periventricular leukomalacia (PVL) was noted in 37 (8.4%) infants. Patent ductus arteriosus (PDA) treatment was given to 115 (16.7%), and retinopathy of prematurity (ROP) treatment was needed in 8.5% of preterm infants.

The study was divided into two periods: period 1 (2016-2019) and period 2 (2020-2022). Mortality was 92 (23.8%) in period 1 vs. 55 (17.6%) in period 2. Morbidities between period 1 vs. period 2 were as follows: BPD 101 (33.8%) vs. 127 (48.1%) (p < 0.0001), PVL 13 (5.7%) vs. 24 (11.2%) (p < 0.038), and sepsis 90 (25.5%) vs. 105 (35.6%) (p = 0.005). Rates of ROP needing treatment and NEC≥2, 18 (7.1%) vs. 23 (10%) and 45 (11.8%) vs. 44 (14.3%), respectively, p=0.239 and p=0.327, respectively, and severe IVH 34 (10%) vs. 23 (8%) (p=0.357).

Conclusion

A significant increase in survival in less than 32 weeks was noted over the study period. Increase in survival was associated with a notable rise in BPD, PVL, and sepsis. Rates of ROP needing treatment and NEC ≥ 2 increased, but the rise didn’t reach statistical significance. On the other hand, a non-statistically significant reduction in severe IVH cases was observed.

## Introduction

Preterm birth remains the leading cause of neonatal mortality and is associated with enduring physical, neurodevelopmental, and socioeconomic consequences [1].

The World Health Organization estimated a global preterm birth rate of 9·9% in 2020, corresponding to 13.4 million preterm live births. From 2010 to 2020, approximately 15% of preterm births occurred before 32 weeks of gestation, which is associated with increased demand for intensive neonatal care [1].

Compared with term infants, preterm infants face markedly higher risks of adverse outcomes, and the likelihood of mortality and morbidity rises as gestational age decreases [2,3].

These outcomes include bronchopulmonary dysplasia (BPD), intraventricular hemorrhage (IVH), periventricular leukomalacia (PVL), necrotizing enterocolitis (NEC), sepsis, and retinopathy of prematurity (ROP), each of which may contribute to long-term disability.

Evidence from multiple neonatal networks demonstrates a clear association between lower gestational age and poorer outcomes. Canadian data from 2022 [4] reported mortality rates of 44% at <24 weeks compared with 2% at 31 weeks, underscoring the critical role of gestational age in survival. 

Gestational age-dependent patterns are also observed for morbidity. A US study [5] reported NEC incidence of 11.5% at 25 weeks, decreasing to 5.4% at 28 weeks.

The Australian and New Zealand Neonatal Network (ANZNN) 2021 study reported a BPD incidence of 89.7% at <24 weeks and 6.8% at 31 weeks [6]. In the same US cohort, PVL and IVH grade ≥3 were 7.8% and 36.4% at 23 weeks, respectively, compared with 2.0% and 5.3% at 28 weeks, respectively, indicating reduced vulnerability of cerebral vasculature with advancing gestation [5].

A similar pattern is observed for ROP requiring treatment; the US study reported rates of 31.8% at 23 weeks and 2.0% at 28 weeks [5]. Likewise, sepsis incidence reported by the Canadian Neonatal Network (CNN) in 2022 was 40% at 24 weeks and 2% at 31 weeks [4].

Published data on preterm infants and short-term morbidities in the United Arab Emirates (UAE) remain limited. Existing studies generally involve small cohorts and provide limited information on very preterm infants [7,8].

One of the earliest UAE reports on preterm mortality by Dawudu et al. evaluated very low birth weight infants (VLBWI) born in Al Ain between 1995 and 1998, reporting mortality rates of 34.5% and 12.2% for infants weighing 500-999 g and 1000-1499 g, respectively [9,10]. Subsequently, Chedid et al. (2004-2006) reported reductions in mortality in these groups at Tawam Hospital (31.3% and 3.6%, respectively) [11]. In 2019, Nusrat et al. reported increased mortality and short-term morbidity among infants weighing 500-1500 g during 2011-2015 compared with 2004-2006 [12].

Many international studies have focused on infants born before 28 weeks’ gestation [5,13]. Although infants born at 28-31 weeks have a lower relative risk, they comprise a larger proportion of preterm births and therefore represent a substantial component of neonatal intensive care admissions [14,3].

In the absence of published UAE data on infants less than 32 weeks of gestation since 2015, we performed a retrospective study at Tawam Hospital, a major tertiary center in the Al Ain region, including infants born between 22 and 31 weeks from 2016 to 2022.

Current data on infants less than 32 weeks of gestation are essential to inform ongoing improvements in neonatal care. Periodic benchmarking against international outcomes can support institutional quality improvement and facilitate informed parental counselling.

## Materials and methods

Aim of the study

To assess the short-term morbidities and mortality of preterm babies less than 32 weeks who were admitted to the neonatal ICU at Tawam Hospital in the period from January 2016 to December 2022.

Study design

This retrospective cohort study includes all infants under 32 weeks of gestation who met the inclusion criteria from the neonatal intensive care unit (NICU) at Tawam Hospital, covering the period from January 1, 2016, to December 31, 2022. Tawam Hospital is a tertiary care center that has a specialized 51-bed level 3 Neonatal Intensive Care Unit (NICU), and admits over 600 infants yearly to the unit.

Inclusion criteria

Preterm infants who were admitted to the NICU and were between 22+0 and 31+6 weeks of gestational age.

Exclusion criteria

We excluded the preterms who had abnormal karyotypes, multiple congenital anomalies incompatible with life, or were outborn babies.

Data collection

We retrospectively collected data on a prespecified data collection sheet. Data collection was done by the principal and co-investigators. A list of admitted patients was obtained from Health Information Management, Tawam Hospital. Health card numbers were used then to retrieve data from the electronic filing system. Data was checked for accuracy and completeness.

The data included infant demographics, maternal complications and interventions, resuscitation practices, peripartum characteristics, and neonatal short-term outcomes until discharge, death, or transfer.

Morbidities recorded included BPD, NEC, late-onset sepsis, IVH, PVL, ROP requiring intervention, patent ductus arteriosus (PDA) needing treatment, and the length of stay of all infants till discharge or death.

Ethics

This cohort study was approved by the Tawam Human Research Ethics Committee, Ethics Committee Approval #MF2058-2022-884.

Statistical analysis

Statistical analysis was done using the BlueSky statistical program version 10.3.2. Numerical data were represented by the mean (standard deviation/range) or median (interquartile range) where appropriate. Categorical data were represented by their respective rates or proportions. The chi-square test was used for categorical variables, and the linear model ANOVA for numerical variables. A p-value of <0.05 was considered statistically significant.

Our study analyzed the outcomes over the seven-year period. We also compared two periods from 2016 to 2019 (period I) and 2020 to 2022 (period II). 

Denominators for short-term outcomes were the following: For BPD, the denominator included those who survived to 36 weeks postmenstrual age (PMA) or beyond. In the case of PDA treatment, it consisted of individuals who lived beyond 24 hours. For PVL, it covered those who were screened by head ultrasound at 4-6 weeks of age. Regarding IVH, the study included infants with IVH ≥ 3 and those who were screened by head ultrasound at one week of age. Late-onset sepsis data were based on patients who survived beyond two days. For ROP requiring intervention, the denominators were those who were screened (≤ 31 weeks or with a birth weight under 1500 grams). Finally, for NEC ≥ 2, the denominator population comprised survivors past 24 hours.

Definitions

Gestational age was determined by the best obstetric estimate or, if unavailable, by the New Ballard score [15]. Gestational age was rounded off to the nearest completed week. Maternal complications included hypertensive disorder complicating pregnancy, gestational diabetes, chorioamnionitis, and premature rupture of membranes (PROM). Hypertensive disorder complicating pregnancy and gestational diabetes were diagnosed according to national guidelines [16,17]. Prolonged rupture of membranes (PROM) is considered when the duration is more than 18 h prior to delivery [18]. Chorioamnionitis diagnosis was determined by the obstetric team, using clinical criteria [19].

Antenatal steroid was recorded as given if at least 1 dose of betamethasone was given within seven days prior to delivery [20]. Perinatal antibiotics were defined as being given in settings where antibiotic prophylaxis is indicated if the following antibiotics are administered intravenously: penicillin, ampicillin, cephalosporins, clindamycin, or vancomycin, and at least a single dose is administered 4 hours prior to delivery [21]. Antenatal magnesium sulfate was considered administered if given within 24 hours prior to delivery [22].

Mode of delivery was recorded as spontaneous vaginal delivery or cesarean section with or without instrumentation. Delayed cord clamping was classified as performed if cord clamping was delayed for 30-60 seconds.

Bronchopulmonary dysplasia was defined using the definition from Jensen et al [23]. This definition classifies bronchopulmonary dysplasia based on the mode of support received at 36 weeks postmenstrual age or at discharge home if earlier. We did not record the grade of BPD. The Papile criteria were used to grade IVH [24], while the Volpe criteria were used to denote PVL [25]. Medical treatment for a patent ductus arteriosus (PDA) was recorded if a complete course of paracetamol or ibuprofen was given with the intention to close the PDA. ROP was defined using the International Classification of Retinopathy of Prematurity 2005 [26], and we recorded ROP requiring intervention in the form of intravitreal injection (anti-vascular endothelial growth factor) or laser photoablation and labeled them as severe ROP in the outcomes assessment. Diagnosis and grading of NEC were performed according to modified Bell criteria [27], and we only recorded cases with NEC stage 2 or more. Late-onset sepsis (hereafter referred to as sepsis throughout the manuscript) was considered present only in infants with positive blood culture. Survival was defined as survival to hospital discharge. 

## Results

Study population

There were a total of 4393 babies admitted to Tawam Hospital during the study period. Of these, 700 infants (16%) were born between 22+0 and 31+6-week gestation, met the inclusion criteria, and were delivered between January 2016 and December 2022. The mean (range) for birth weight and gestational age was 1113 grams (350-2260) and 27 weeks (31-22), respectively, from 2016 to 2022. Overall, 392 of 700 infants (56.0%) were male, and 308 of 700 (44.0%) were female. Additionally, 571 of 700 infants (82%) were born to Emirati families, 270 of 687 (39%) were conceived via in vitro fertilization, and 315 of 697 deliveries (45.2%) were multiple births (Table 1).

**Table 1:** Characteristics of infants in the study group from 2016 to 2022 The chi-square test was used for analysis of categorical variables. Linear Model ANOVA was used for analysis of numerical variables. A p-value < 0.05 was considered statistically significant. *Statistically significant p-values. ^F-Values. ^X^ Chi-Square Value.

Variable	2016	2017	2018	2019	2020	2021	2022	Total	P-value	Test Statistical Value
Number	95	118	90	84	94	116	103	700		
Birth weight in grams, mean (range)	1106 (420-2055)	1066 (400-1810)	1119 (350-2075)	1221 (505-2015)	1120 (350-2145)	1111 (400-2060)	1071 (360-2260)	1113 (350-2260)	0.213	1.39^
Gestational age in weeks, mean (range)	27 (22-31)	27 (22-31)	28(22-31)	28(23-31)	28(22-31)	28(22-31)	27(22-31)	27(22-31)	0.09	1.83^
Emirati Nationals n/N (%)	82/95 (86)	97/118 (82)	73/90 (81)	79/83 (95)	76/94 (81)	88/116 (76)	76/103 (74)	571/700 (82)	0.005*	18.47^ x^
Female n/N (%)	36/95 (38)	47/118 (40)	50/90 (56)	42/84 (50)	41/94 (44)	46/116 (40)	46/103 (45)	308/700 (44)	0.158	9.28^x^
Invitro Fertilization n/N (%)	46/91 (50)	57/115 (50)	41/90 (46)	31/83 (37)	30/93 (32)	33/115 (29)	32/100 (32)	270/687 (39)	<0.001*	39.67^ x^
Multiple Birth n/N (%)	46/93 (49)	63/118 (53)	51/90 (57)	42/83 (51)	34/94 (36)	44/116 (37)	33/103 (32)	315/697 (45)	<0.001*	40.03^ x^

Perinatal complication and interventions

**Figure 1:**
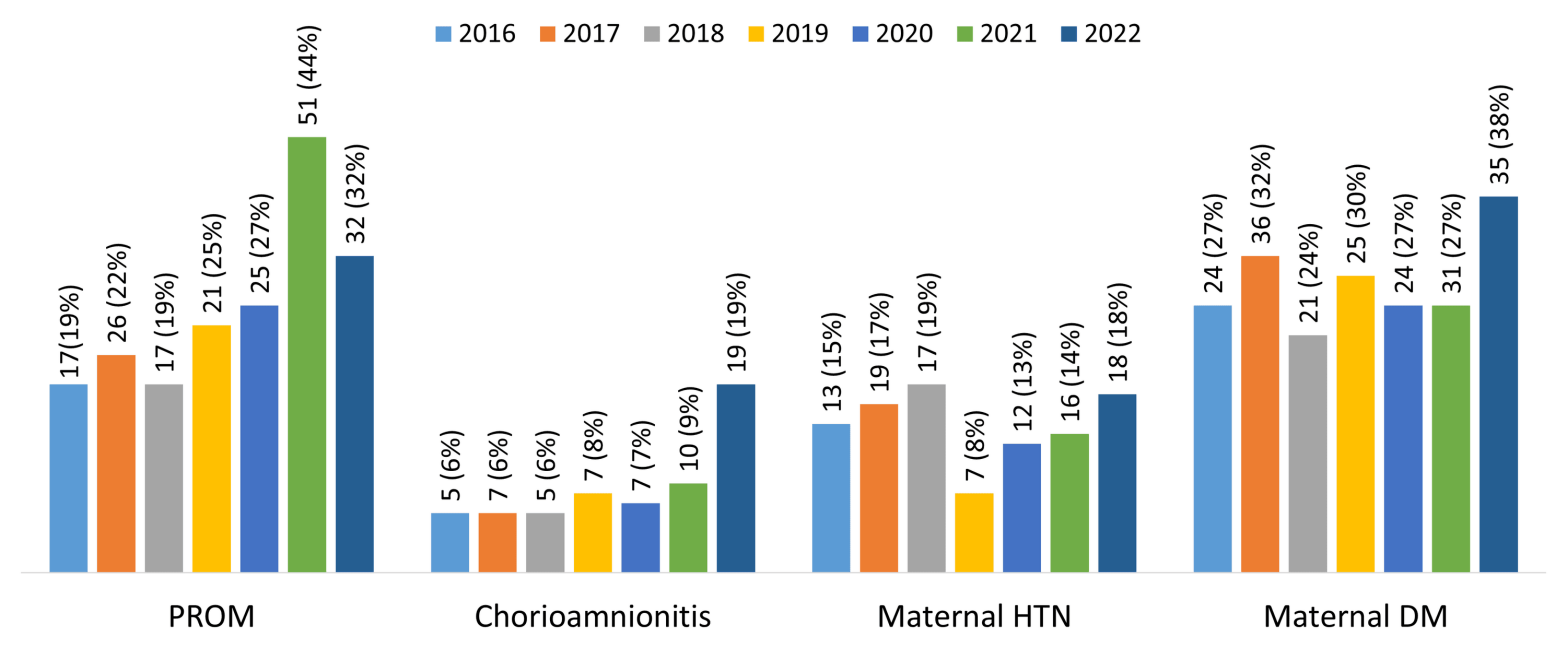
Trends in maternal complications of the infants born during the study period 2016-2022 HTN: Hypertension; DM: Diabetes Mellitus

Over the seven-year study period, the incidence of premature rupture of membranes, maternal chorioamnionitis, maternal hypertension, and maternal diabetes mellitus was 189 of 690 (27%), 60 of 681 (9%), 102 of 681 (15%), and 196 of 688 (29%), respectively (Figure 1).

**Figure 2:**
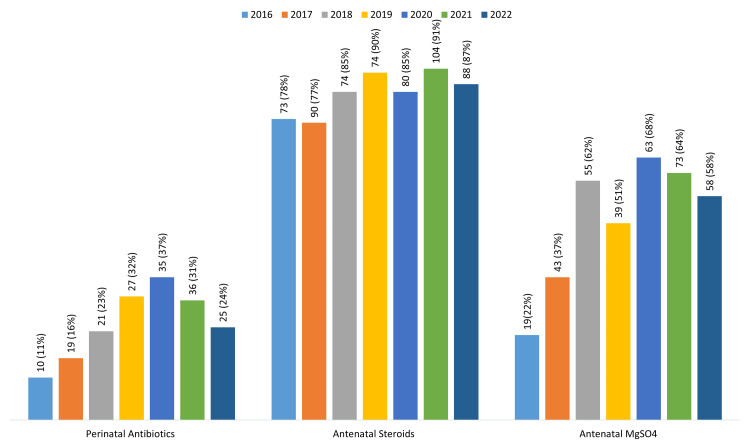
Trends in antenatal interventions of the infants born during the study period 2016-2022 MgSO4: Magnesium Sulfate

Perinatal interventions, including perinatal antibiotics, antenatal corticosteroids, and antenatal magnesium sulfate, were administered in 173 of 698 cases (25%), 583 of 688 (85%), and 350 of 676 (53%), respectively, over the study period. Use of antenatal corticosteroids increased steadily over time, from 78.0% in 2016 to 87.0% in 2022 (Figure 2).

Peripartum care practices

Most preterm infants in the study population were delivered by cesarean section (467 of 700 [67%]). Use of assisted delivery devices was rare, occurring in 2 of 695 infants (0.3%) born at less than 32 weeks’ gestation (Table 2).

**Table 2:** Peripartum characteristics of the infants born during the study period 2016-2022 The chi-square test was used for analysis. A p-value < 0.05 was considered statistically significant. *Statistically significant p-values. ^X^ Chi-Square Value

Variable	2016	2017	2018	2019	2020	2021	2022	Total	p-value	Test Statistical Value
Spontaneous Vaginal Delivery n/N (%)	30/95 (32%)	55/118 (47%)	28/90 (31%)	17/84 (20%)	28/94 (30%)	41/116 (35%)	34/103 (33%)	233/700 (33%)	0.01*	16.9^ x^
Instrumentation n/N (%)	0/95 (0%)	0/118 (0%)	0/90 (0%)	1/84 (1%)	0/93 (0%)	0/115 (0%)	1/100 (1%)	2/695 (0.3%)	0.466	5.62^ x^

Survival

The overall survival rate was 553 of 700 infants (79.0%) (Table 3). Mortality decreased with increasing gestational age, from 100.0% (36/36) at 22 weeks’ gestation to 3.2% (5/157) at 31 weeks (Table 4). Mortality declined from 92 of 387 infants (23.8%) in 2016-2019 (Period 1) to 55 of 313 (17.6%) in 2020-2022 (Period 2) (p = .045) (Table 5). Additionally, survival among infants born at less than 28 weeks’ gestation increased from 80/156 (51.3%) to 85/133 (63.9%) between the two periods (Table 6).

**Table 3:** Outcomes across the years BPD: bronchopulmonary dysplasia; NEC: necrotizing enterocolitis; PVL: periventricular leukomalacia; IVH: intraventricular leukomalacia; PDA: patent ductus arteriosus; ROP: retinopathy of prematurity; IQR: interquartile range

Year	BPD any grade n/N (%)	NEC≥2 n/N (%)	Sepsis n/N (%)	PVL n/N (%)	IVH≥3 n/N (%)	PDA treatment n/N (%)	ROP needing treatment n/N (%)	Mortality prior to discharge n/N (%)	Length Of Stay in days, All Cases, Median (IQR)
2016	27/71 (38.0)	14/92 (15.2)	29/85 (34.1)	6/50 (12.0)	10/78 (12.8)	19/92 (20.7)	1/61 (1.6)	26/95 (27.4)	49 (25.5, 87)
2017	23/85 (27.1)	6/118 (5.1)	25/104 (24)	2/72 (2.8)	15/102 (14.7)	15/118 (12.7)	3/77 (3.9)	32/118 (27.1)	43.5 (31, 78.5)
2018	19/70 (27.1)	15/87 (17.2)	17/85 (20.0)	1/54 (1.9)	5/76 (6.6)	5/87 (5.7)	4/59 (6.8)	22/90 (24.4)	51.5 (29.25, 76)
2019	32/73 (43.8)	10/84 (11.9)	19/79 (24.1)	4/51 (7.8)	4/79 (5.1)	13/84 (15.5)	10/58 (17.2)	12/84 (14.3)	55.5 (37.25, 90.5)
2020	33/81 (40.7)	8/94 (8.5)	27/91 (29.7)	8/69 (11.6)	9/89 (10.1)	22/94 (23.4)	7/70 (10.0)	14/94 (14.9)	64 (34, 82)
2021	46/95 (48.4)	17/115 (14.8)	43/108 (39.8)	6/76 (7.9)	6/105 (5.7)	22/115 (19.1)	4/83 (4.8)	23/116 (19.8)	57.5 (36, 91)
2022	48/88 (54.5)	19/98 (19.4)	35/96 (36.5)	10/69 (14.5)	8/93 (8.6)	19/97 (19.6)	12/76 (15.8)	18/103 (17.5)	64 (33, 99.5)
Total	228/563 (40.5)	89/688 (12.9)	195/648 (30.1)	37/441 (8.4)	57/622 (9.2)	115/687 (16.7)	41/484 (8.5)	147/700 (21.0)	55.5 (32, 85.25)

**Table 4:** Outcomes by gestational age BPD: bronchopulmonary dysplasia; NEC: necrotizing enterocolitis; PVL: periventricular leukomalacia; IVH: intraventricular leukomalacia; PDA: patent ductus arteriosus; ROP: retinopathy of prematurity

Gestational Age / Outcome	22 weeks, n/N (%)	23 weeks, n/N (%)	24 weeks, n/N (%)	25 weeks, n/N (%)	26 weeks, n/N (%)	27 weeks, n/N (%)	28 weeks, n/N (%)	29 weeks, n/N (%)	30 weeks, n/N (%)	31 weeks, n/N (%)
Mortality prior to discharge	36/36 (100.0)	47/54 (87.0)	17/39 (43.6)	10/46 (21.7)	9/47 (19.1)	5/67 (7.5)	4/79 (5.1)	6/72 (8.3)	8/103 (7.8)	5/157 (3.2)
PDA treatment	0/30 (0.0)	13/50 (26.0)	23/39 (59.0)	25/45 (55.6)	16/47 (34.0)	11/67 (16.4)	16/79 (20.3)	4/72 (5.6)	3/101 (3.0)	4/157 (2.5)
NEC≥2	2/30 (6.7)	16/50 (32.0)	18/39 (46.2)	10/45 (22.2)	7/47 (14.9)	15/67 (22.4)	4/79 (5.1)	2/72 (2.8)	8/102 (7.8)	7/157 (4.5)
BPD (any grade)	0/0 (0)	10/10 (100.0)	22/24 (91.7)	33/38 (86.8)	27/38 (71.1)	40/63 (63.5)	34/74 (45.9)	21/66 (31.8)	22/97 (22.7)	19/153 (12.4)
PVL	1/1 (100.0)	3/12 (25.0)	7/24 (29.2)	6/39 (15.4)	5/42 (11.9)	5/62 (8.1)	2/75 (2.7)	1/66 (1.5)	1/62 (1.6)	6/58 (10.3)
IVH≥3	10/11 (90.9)	11/29 (37.9)	10/33 (30.3)	6/43 (14.0)	3/45 (6.7)	7/65 (10.8)	4/78 (5.1)	2/69 (2.9)	1/99 (1.0)	3/150 (2.0)
ROP needing treatment	0/0 (0)	9/14 (64.3)	12/28 (42.9)	6/39 (15.4)	5/41 (12.2)	7/63 (11.1)	1/76 (1.3)	0/67 (0.0)	0/97 (0.0)	1/59 (1.7)
Sepsis	6/17 (35.3)	18/33 (54.5)	26/36 (72.2)	31/44 (70.5)	25/47 (53.2)	22/66 (33.3)	14/78 (17.9)	14/72 (19.4)	19/100 (19.0)	20/155 (12.9)

**Table 5:** Outcomes between two periods 2016-2019 vs. 2020-2022 The chi-square test was used for analysis of categorical variables. Linear Model ANOVA was used for analysis of numerical variables. A p-value < 0.05 was considered statistically significant. *Statistically significant p-values. ^F-Values. ^X^ Chi-Square Value BPD: bronchopulmonary dysplasia; NEC: necrotizing enterocolitis; PVL: periventricular leukomalacia; IVH: intraventricular leukomalacia; PDA: patent ductus arteriosus; ROP: retinopathy of prematurity; IQR: interquartile range

Outcome	Period I 2016-2019 (Total 387) n/N (%)	Period II 2020-2022 (Total 313) n/N (%)	Total n/N (%)	p-value	Test Statistical Value
BPD (any grade)	101/299 (33.8)	127/264 (48.1)	228/563 (40.5)	< 0.001*	11.94^ x^
NEC≥2	45/381 (11.8)	44/307 (14.3)	89/688 (12.9)	0.327	0.95^ x^
Sepsis	90/353 (25.5)	105/295 (35.6)	195 (30.1)	0.005*	7.78^ x^
PVL	13/227 (5.7)	24/214 (11.2)	37/441 (8.4)	0.038*	4.31^ x^
IVH≥3	34/355 (10.1)	23/287 (8.0)	57/622 (9.2)	0.357	0.84^ x^
PDA treatment	52/381 (13.6)	63/306 (20.6)	115/687 (16.7)	0.015*	5.86^ x^
ROP needing treatment	18/255 (7.1)	23/229 (10.0)	41/484 (8.5)	0.239	1.38^ x^
Mortality prior to discharge	92/387 (23.8)	55/313 (17.6)	147/700 (21.0)	0.045*	4.01^ x^
Length of Stay (days), All Cases, Median (IQR)	52.000 (29.500, 81.000)	63.000 (34.000, 94.000)	55.500 (32.000, 85.250)	0.046*	3.98^
Length of Stay, Survivors, Median (IQR)	63.000 (40.000, 89.000)	68.500 (44.250, 102.250)	67.000 (42.000, 95.000)	0.133	2.26^

**Table 6:** Survival and outcomes in the extreme preterm population between 2016-2019 vs. 2020-2022 The chi-square test was used for analysis. A p-value < 0.05 was considered statistically significant. *Statistically significant p-values ^X^ Chi-Square Value. BPD: bronchopulmonary dysplasia; NEC: necrotizing enterocolitis; PVL: periventricular leukomalacia; IVH: intraventricular leukomalacia; ROP: retinopathy of prematurity

Outcomes	Period I 2016-2019 (Total 156) n/N (%)	Period II 2020-2022 (Total 133) n/N (%)	p-value	Test Statistical Value
Survival	80/156 (51.3)	85/133 (63.9)	0.031*	4.67^X^
NEC≥2	38/150 (25.3)	30/128 (23.4)	0.714	0.13^ X^
BPD (any grade)	56/83 (67.5)	76/90 (84.4)	0.009*	6.88^ X^
PVL	6/83 (7.2)	21/97 (21.6)	0.007*	7.2^ X^
IVH≥3	27/115 (23.5)	20/111 (18.0)	0.312	1.02^ X^
ROP needing treatment	16/91 (17.6)	23/94 (24.5)	0.251	1.31^ X^
Sepsis	62/125 (49.6)	66/118 (55.9)	0.323	0.97^ X^

Morbidities

Bronchopulmonary dysplasia (BPD) occurred in 228 of 563 infants (40.5%), increasing with decreasing gestational age from 19 of 153 (12.4%) at 31 weeks to 10 of 10 (100.0%) at 23 weeks. The incidence of BPD also increased from 101 of 299 (33.8%) in Period 1 to 127 of 264 (48.1%) in Period 2 (p < .001).

Intraventricular hemorrhage (IVH) of grade 3 or higher occurred in 57 of 622 infants (9.2%), decreasing with advancing gestational age from 10 of 11 (90.9%) at 22 weeks to 3 of 150 (2.0%) at 31 weeks. There was no significant difference between Period 1 (34 of 355 [10.1%]) and Period 2 (23 of 287 [8.0%]) (p = .357).

Periventricular leukomalacia (PVL) was identified in 37 of 441 infants (8.4%), peaking at 24 weeks (7 of 24 [29.2%]) and increasing from 13 of 227 (5.7%) in Period 1 to 24 of 214 (11.2%) in Period 2 (p = .038).

Patent ductus arteriosus (PDA) treatment was administered in 115 of 687 infants (16.7%), peaking at 24 weeks (23 of 39 [59.0%]) and increasing from 52 of 381 (13.6%) in Period 1 to 63 of 306 (20.6%) in Period 2 (p = .015).

Necrotizing enterocolitis (NEC) occurred in 89 of 688 infants (12.9%), decreasing with increasing gestational age from 18 of 39 (46.2%) at 24 weeks to 7 of 157 (4.5%) at 31 weeks. The incidence did not differ significantly between Period 1 (45 of 381 [11.8%]) and Period 2 (44 of 307 [14.3%]) (p = .327).

Severe retinopathy of prematurity (ROP) occurred in 41 of 484 infants (8.5%), highest at 23 weeks (9 of 14 [64.3%]) and decreasing to 1 of 59 (1.7%) at 31 weeks. No significant change was observed between Period 1 (7.1%) and Period 2 (10.0%) (p = .239).

Sepsis occurred in 195 of 648 infants (30.1%), highest at 24 weeks (26 of 36 [72.2%]) and lowest at 31 weeks (20 of 155 [12.9%]). The incidence increased from 90 of 353 (25.5%) in Period 1 to 105 of 295 (35.6%) in Period 2 (p = .005).

The median length of stay was 55.5 days, with no significant difference among survivors between Period 1 (63.0 days; IQR, 40.0-89.0) and Period 2 (68.5 days; IQR, 44.3-102.3) (p = .133) (Tables 3-5).

## Discussion

Outcomes were compared between two periods at Tawam Hospital (2016-2019 and 2020-2022; Table 5). Mortality decreased significantly in the later period, while the length of stay increased. Rates of BPD, NEC, sepsis, PVL, PDA treatment, and ROP needing treatment were higher in the more recent cohort.

The increased morbidity observed may reflect improved survival of extremely preterm infants. A greater proportion of extremely preterm infants survived in period II compared with period I (Table 6). Advances in clinical practices and diagnostic technologies over time may have further contributed to these findings.

Moreover, outcomes of infants born at <32 weeks’ gestation at Tawam Hospital were compared with those reported by CNN 2022 [4], ANZNN 2022 [6], Neonatal Research Network of Japan (NRNJ) 2018 [28], and King Faisal Specialist Hospital (KFSH) 2020 [29]. Significant variability in outcomes was observed across networks. During the period from 2016 to 2022, Tawam Hospital demonstrated higher reported rates of bronchopulmonary dysplasia, necrotizing enterocolitis, sepsis, periventricular leukomalacia, and mortality compared with the referenced networks. Rates of retinopathy of prematurity requiring treatment were also relatively high at Tawam Hospital; however, the highest rate was reported by NRNJ (Table 7).

**Table 7:** The data presented in the table encompasses findings from multiple hospitals and studies, spanning different years and focusing on outcomes among ≤ 32-week premature infants Denominators for Tawam Hospital: For BPD, any grade denominators = those who survived 36-week PMA and beyond. For PDA treatment, denominators = all who survived 24 hours. For PVL denominators = all who got screened at 4 weeks of age. For IVH≥3, denominators = all who got screened by 1 week of age. For sepsis, denominators = all who survived beyond 2 days. For ROP needing treatment, denominators=all who got screened. For NEC≥2 denominators= all who survived 24 hours. BPD: bronchopulmonary dysplasia; NEC: necrotizing enterocolitis; PVL: periventricular leukomalacia; IVH: intraventricular leukomalacia; PDA: patent ductus arteriosus; ROP: retinopathy of prematurity; CNN: Canadian Neonatal Network; ANZNN: Australian and New Zealand Neonatal Network; NRNJ: Neonatal Research Network Japan; KFSH: King Faisal Specialist Hospital; N.P.: Not Published

Outcomes	2016-2022 Tawam Hospital Total infants 700, 22-31 weeks n/N (%)	2022 CNN[4] Total infants 4173, <33 weeks n/N (%)	2022 ANZNN[6] Total Infants 3406, <32weeks n/N (%)	2018 NRNJ [14] Total Infants 4213, 22-32 weeks n/N (%)	2020 KFSH [29] Total 528, 23-31 weeks n/N (%)
BPD any grade	228/563 (40.5)	1225/3891 (31.0)	1171/3102 (37.7)	852/4020 (21.2)	129/528 (24.4)
NEC≥2	89/688 (12.9)	174/4173 (4.0)	134/3271 (3.4)	168/4213 (4.0)	48/528 (9.1)
Sepsis	195/648 (30.1)	426/4173 (10.0)	248/1096 (22.6)	368/4193 (8.8)	99/528 (18.8)
PVL	37/441 (8.4)	248/3576 (7.0)	73/2346 (3.1)	129/4188 (3.1)	30/528 (5.7)
IVH≥3	57/622 (9.2)		171/3131 (5.4)	180/4188 (4.3)	57/528 (10.8)
PDA treatment	115/687 (16.7)	-	--	1739/3716 (46.8)	-
ROP needing treatment	41/484 (8.5)	137/2012 (7.0)	N. P	680/3887 (17.5)	16/528 (3)
Mortality prior to discharge	147/700 (21.0)	276/4173 (7.0)	261/3406 (8)	202/4213 (4.6)	106/528 (20.0)

Differences in infant and maternal characteristics across networks may partly explain the observed variation in outcomes and warrant further analysis. The study population at Tawam Hospital had one of the highest proportions of multiple births compared with other networks. In addition, the CNN 2022 and NRNJ 2018 cohorts included infants born at 32 weeks’ gestation, which may influence outcome comparisons. Compared with Tawam Hospital, ANZNN 2022 included a markedly lower representation of infants born at ≤24 weeks among those <32 weeks gestation. Given that infants born at 22-24 weeks are at the highest risk for major morbidities, these differences necessitate cautious interpretation. Furthermore, the KFSH 2020 study excluded infants born at <23 weeks and included only infants weighing >400 g when reporting short-term morbidities among those <32 weeks’ gestation, potentially affecting reported morbidity and mortality rates.

In summary, the data highlights significant variability in medical outcomes among premature infants across different networks, gestational ages, and years. This underscores the importance of ongoing research and standardization in neonatal care to improve outcomes and reduce complications in this vulnerable population.

Strengths

Strengths of this study include the large cohort of extremely preterm infants from a level III tertiary NICU, gestational age-stratified analyses of morbidity and mortality to support more precise counseling and outcome prediction, and the evaluation of temporal trends in outcomes.

Limitations

Data were collected from a single tertiary center and analyzed retrospectively, with long-term outcomes unavailable.

## Conclusions

A significant improvement in overall preterm survival was observed over time, with the survival gains being more pronounced among extremely preterm infants. This increase in survival may have unmasked a higher burden of morbidity, particularly neurological and pulmonary complications. These findings underscore the ongoing challenge in the care of extremely preterm infants of balancing improvements in survival with quality-of-survival outcomes. Larger multicenter studies with greater sample sizes are needed to define national outcome benchmarks.
